# Preparation, Reliability, and Validity of a Japanese Version of the “Satisfaction of Treatment among Caregivers of Dependent Type 2 Diabetic Patients” Questionnaire

**DOI:** 10.1155/2020/4960130

**Published:** 2020-06-15

**Authors:** Satoshi Ida, Ryutaro Kaneko, Kanako Imataka, Kaoru Okubo, Yoshitaka Shirakura, Kentaro Azuma, Ryoko Fujiwara, Hiroka Takahashi, Kazuya Murata

**Affiliations:** Department of Diabetes and Metabolism, Ise Red Cross Hospital, 1-471-2 Funae, 1-Chome, Ise-shi, Mie 516-8512, Japan

## Abstract

The aim of this study was to prepare a Japanese version of the “Satisfaction of Treatment among Caregivers of Dependent Type 2 Diabetic Patients” (STCD_2_-J) questionnaire, which is used to assess the satisfaction of family caregivers with respect to the treatment for elderly patients with type 2 diabetes mellitus who require support. In addition, the reliability and validity of the STCD_2_-J questionnaire were analyzed. A Japanese version of the original STCD_2_ questionnaire was prepared, revised, and back-translated; the back-translated version was sent to the authors of the original version for confirmation. Family caregivers of patients with type 2 diabetes mellitus aged ≥65 years who regularly underwent medical examinations at the diabetes mellitus outpatient clinic of Ise Red Cross Hospital were included. Cronbach's *α* coefficient was calculated to assess internal consistency. Exploratory factor analyses were performed to assess construct validity, and Pearson's correlation coefficients between STCD_2_-J score and HbA1c as well as the degree of satisfaction with patients' blood glucose levels, depression, and negative self-assessment of nursing care were calculated to assess criterion-related validity. This study included 208 individuals (55 males and 153 females). Cronbach's *α* coefficient was 0.88. Factor analyses showed a single-factor structure both with and without rotation. The STCD_2_-J scores were significantly inversely correlated with HbA1c (*r* = −0.27, *P* < 0.001). Significant correlations were observed between the STCD_2_-J scores and degree of satisfaction with patients' blood glucose levels (*r* = 0.43, *P* < 0.001), depression (*r* = −0.20, *P* = 0.003), and negative self-assessment of nursing care (*r* = −0.19, *P* = 0.004). The reliability and validity of the STCD_2_-J questionnaire were confirmed. The STCD_2_-J questionnaire can be used in Japan as a tool to assess the satisfaction of family caregivers with the treatment of elderly patients with type 2 diabetes mellitus requiring support.

## 1. Introduction

As the elderly population increases, the number of elderly patients with diabetes mellitus also increases [1, 2]. In addition to a high prevalence of microvascular complications and macroangiopathy, elderly patients with diabetes mellitus often have cognitive impairment or reduced activity of daily living (ADL) [3]. In these elderly patients with diabetes mellitus, self-management of diet therapy, exercise therapy, and pharmacotherapy is often difficult; family support is required [4, 5]. In addition to a large number of antidiabetic drugs and complicated drug administration methods for elderly patients with diabetes mellitus, there is a need to pay attention to possible side effects, such as hypoglycemia [3, 6, 7]. Given the growing number of elderly patients with diabetes mellitus who require support, the roles of family caregivers in the treatment of diabetes mellitus will be essential in the future.

Treatment satisfaction in patients is one of the most important outcomes in daily clinical practice and has been emphasized in the treatment of diabetes mellitus [8]. However, considering the important roles of family caregivers, their satisfaction with the treatment of elderly patients with diabetes mellitus requiring support may also be important. Previous studies have reported that decreased satisfaction and quality of life (QOL) among caregivers were associated with the caregivers' mental health problems, such as depression [9], and with nursing care burden and burnout of caregivers [10–12]. Moreover, caregiver satisfaction with the treatment of patients with diabetes mellitus has been reported to be closely associated with effective glycemic control in patients [13, 14] and the maintenance of patient QOL [15]. These results suggest that the family caregiver's high level of satisfaction regarding the treatment of dependent diabetic patients is believed to affect not only the maintenance of the caregiver's own health but also the good glycemic control and QOL of the patient. Therefore, the degree of satisfaction among family caregivers of the treatment of elderly patients with type 2 diabetes mellitus requiring support is clinically important.

Currently, there is no tool to evaluate the satisfaction of family caregivers with the treatment of type 2 diabetic elderly patients who require support, although evaluative parental satisfaction tools that gauge the treatment of pediatric patients with type 1 diabetes have been developed in previous studies [16, 17]. The Satisfaction of Treatment among Caregivers of Dependent Type 2 Diabetic Patients (STCD_2_) questionnaire is a self-administered questionnaire that was developed to measure the satisfaction of caregivers with respect to the treatment of patients with type 2 diabetes mellitus in Spain [13]. The STCD_2_ questionnaire comprises 7 questions related to the degree of satisfaction, including the treatment for patients with diabetes mellitus, patients' acceptance of treatment, ease of treatment, number of treatments, knowledge regarding antidiabetic treatment, continuation of treatment, and recommendation of treatment to others. In the article reporting the original STCD_2_ development, Cronbach's alpha of 0.93 and intraclass correlation coefficient of 0.96 were reported in the target population of dependent patients with type 2 diabetes. Moreover, significant correlations between the STCD_2_ questions and the results of HbA1c and patients' satisfaction regarding blood glucose levels (*r* = −0.24 to −0.35 and *r* = 0.40–0.86, respectively) were reported, demonstrating construct validity, and the STCD_2_ was reported to be a valuable tool.

The STCD_2_ questionnaire has not been translated into Japanese, and its reliability and validity have not been verified. The rate of aging in Japan is remarkably higher than that in other countries [18]. In addition, the social security system and family structure differ among countries, and there are racial and cultural differences in the approaches used to support families [19, 20]. These are important factors to be considered in the preparation of a translated version for family caregivers of elderly patients with diabetes mellitus in Japan. Therefore, the aim of this study was to prepare a Japanese version of the Satisfaction of Treatment among Caregivers of Dependent Type 2 Diabetic Patients (STCD_2_-J) questionnaire for family caregivers of elderly patients with type 2 diabetes mellitus who require support in Japan. In addition, the reliability and validity of the STCD_2_-J questionnaire were determined.

## 2. Materials and Methods

### 2.1. Translation

The STCD_2_ questionnaire comprises 7 questions regarding the degree of satisfaction, including current treatments for patients with diabetes mellitus, the degree of acceptance of treatment, ease of treatment (e.g., injections and oral medications), number of treatments given per day, knowledge regarding antidiabetic treatment, continuation of treatment, and recommendation of current treatments to other patients [13]. Responses were rated on a 5-point scale ranging from 1 (very satisfied) to 5 (completely not satisfied), with 5 points given for a rating of 1 and 1 point given for a rating of 5 for each question. The total score ranges from 7 to 35, with a higher score indicating a greater degree of satisfaction with patient treatment. The Japanese translation of the original STCD_2_ questionnaire was administered independently by 2 members of our group (SI and RK). The translated document was revised by discussing each question within our group. Next, the Japanese translation was back-translated to English by a native speaker. The back-translated version was sent to the authors of the original STCD_2_ questionnaire, who confirmed that there were no issues compared with the original version. In June 2018, consent from the authors of the original STCD_2_ questionnaire was obtained to allow the preparation of the STCD_2_-J version.

### 2.2. Study Design and Subjects

This study involved family caregivers of elderly patients with type 2 diabetes mellitus requiring treatment support who regularly underwent medical examinations at the diabetes mellitus outpatient clinic of Ise Red Cross Hospital in Ise City, Mie Prefecture. This study was conducted after written consent was obtained from participants and after approval was obtained from the Institutional Review Board of Ise Red Cross Hospital. Eligibility criteria included families of patients aged ≥65 years requiring treatment support who were medically examined at our diabetes mellitus outpatient clinic between December 2018 and April 2019. In addition, the families had to be involved in the treatment of patients with type 2 diabetes mellitus. When patients and their family members visited the clinic, the attending physician or staff provided explanations about the study, and only those giving consent participated. The family members who did not accompany the patient were asked through telephone to confirm whether they could visit the clinic at a later date, and they were invited to participate. The patients and family members were asked to answer the questionnaire after giving consent. The questionnaire was used as a reference to determine whether the family members provided support for the patient. In the questionnaire, the family members were asked, “Do you provide support to the patient with regard to diet, exercise, diabetes medications, and psychological aspects? ” Those who responded “yes” when asked to provide a “yes” or “no” response were classified as family caregivers. The exclusion criteria involved individuals with a history of a diagnosis of alcoholism, severe psychiatric disorders, or malignancies and individuals who were unable to participate in the study of their own volition. The Dementia Assessment Sheet for Community-based Integrated Care System 8-items (DASC-8) developed by Toyoshima et al. [21] was used to define elderly patients with diabetes mellitus requiring support, and the criteria for the total DASC-8 score were set at ≥11 points. The DASC-8 is a questionnaire comprising 8 questions related to cognitive function or ADL rated on a four-point scale, with 1–4 points allocated to each question. The total score ranges from 8 to 32; the higher the score, the lower the cognitive function and ADL.

### 2.3. Measurement of Variables

Age, sex, relationship with the patient (degree relative), occupation, duration of providing support to the patient, training experience with courses and educational programs on diabetes mellitus, presence or absence of other individuals living in the same household, number of chronic diseases, self-assessment of support provided, degree of satisfaction with the blood sugar level of the patient, and signs of depression were evaluated as background factors for family caregivers. Age, diabetes mellitus type, HbA1c, insulin use, microangiopathy, and macroangiopathy were evaluated as background factors for the patients. The Japanese version of the Patient Health Questionnaire 9 (J-PHQ-9) [22], which comprises 9 items, was used to measure depression. Responses were evaluated using a 4-point scale for symptoms in the past 2 weeks (almost every day = 3 points, about half of the time = 2 points, a few days = 1 point, and not at all = 0 points). The total score ranges from 0 to 27, with a higher score indicating a higher severity of depression. A single-item questionnaire was administered to assess the satisfaction of the caregivers with the patients' blood glucose levels [13]. The question was “are you satisfied with the blood sugar level of the patient you are supporting or providing nursing care for?” Responses were provided on a 5-point scale from “very satisfied” to “not satisfied at all.” The negative rating scale of the cognitive appraisal of caregiving scale (CACS) developed by Hirose et al. [23] was used in the self-assessment of support. The CACS is a questionnaire that comprises 13 items related to social awkwardness, anxiety regarding continuing nursing care, and feeling a psychological burden with relationships. Responses are rated on a 4-point scale from “I do not think that is the case at all” to “I absolutely think so”; 1–4 points are allocated to each question. The total score ranges from 13 to 72; the higher the score, the more negative the self-assessment.

### 2.4. Statistical Analysis

The background factors of the participants and the score distribution of the questionnaire were described. Cronbach's *α* coefficient was calculated to evaluate internal consistency. Exploratory factor analyses (principal factor method, varimax rotation, and promax rotation) were also performed to evaluate construct validity and verify the number of factors for the STCD_2_-J questionnaire. Pearson's correlation coefficients between the total STCD_2_-J scores and item-specific STCD_2_-J scores and HbA1c as well as the family caregivers' degree of satisfaction with blood glucose levels of the patient were calculated to evaluate construct validity. Decreases in caregiver satisfaction with patient treatment and QOL have been reported to be associated with depression or negative self-assessment of nursing care [9, 24, 25]. Therefore, Pearson's correlation coefficients between the STCD_2_-J and J-PHQ-9 and CACS scores were also determined in the evaluation of criterion-related validity. Our hypotheses were that (1) STCD_2_-J scores would inversely correlate with HbA1c levels and J-PHQ-9 and CACS scores, and that (2) STCD_2_-J scores would positively correlate with the family caregivers' satisfaction regarding the patients' blood glucose levels. In addition, we hypothesized that STCD_2_-J items 3 “satisfaction with ease of injection and oral administration” and 4 “satisfaction with the number of treatments administered per day” would have significant inverse correlations with the actual number of oral hypoglycemic agents taken and the number of insulin injections administered to patients per day. Therefore, these variables were also analyzed. The (two-sided) significance level was set as <0.05, and STATA version 16.0 (Stata Corporation LP, College Station, TX) was used for the analyses.

## 3. Results

In total, 208 individuals (55 males and 153 females) who met the eligibility criteria were included in this study. The background characteristics of the participants are shown in Table 1. The mean age of family caregivers was 64 years, and females made up 73% of the participants. The majority of the family caregivers were spouses of the patients, and the mean duration of providing support for the patients was 10 years. Family caregivers had a mean of 1.3 chronic diseases, and 48% were employed. The majority of patients with diabetes mellitus were elderly males; the mean age was 72 years, and 55% of them were males. Insulin use was also reported in 70% of patients.

**Figure 1:**
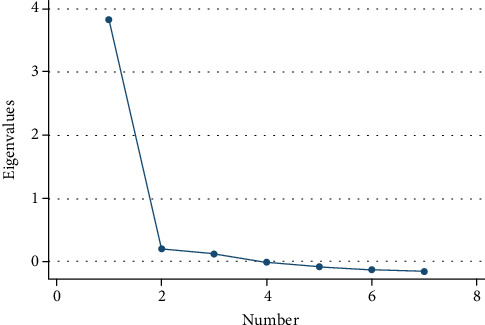
Screen plot.

The results of item-specific STCD_2_-J scores and total scores, factorial loading, and Cronbach's *α* coefficients are presented in Table 2. The total STCD_2_-J score was 24.9 points, and Cronbach's *α* coefficient was 0.88, which demonstrated reliability. The Kaiser-Meyer-Olkin index was 0.86, which indicated a reasonable sample size for factorial analyses. The factor loads for the STCD_2_-J questionnaire without rotation calculated using the principal factor method ranged from 0.45 to 0.82 (eigenvalue of the first factor = 3.8), which was considered to show a single-factor structure. Even after using varimax rotation and promax rotation, interpretable factors could not be identified, and screen plots (Figure 1) suggested that there was a single-factor structure.

For the assessment of criterion-related validity, the total STCD_2_-J score (*r* = −0.27, *P* < 0.001) and item-specific STCD_2_-J scores, except the score for item 5 (*r* = from − 0.13 to − 0.29), were significantly correlated with HbA1c. There were also significant correlations between the STCD_2_-J scores and degree of satisfaction with patients' blood glucose levels (*r* = 0.43, *P* < 0.001), depression (*r* = −0.20, *P* = 0.003), and negative self-assessment of nursing care (*r* = −0.19, *P* = 0.004). The correlation coefficients between STCD_2_-J items 3 and 4 and the number of OHAs taken by the patients were *r* = −0.004 (*P* = 0.969) and *r* = 0.101 (*P* = 0.337), respectively. The correlation coefficients between STCD_2_-J items 3 and 4 and the number of insulin injections administered to patients were *r* = −0.30 (*P* = 0.004) and *r* = −0.34 (*P* = 0.001), respectively.

## 4. Discussion

In this study, the STCD_2_-J questionnaire was prepared to assess the satisfaction of family caregivers with the treatment of elderly patients with type 2 diabetes mellitus who require support in Japan; the reliability and validity of the tool were analyzed. The results confirmed the reliability and validity of the STCD_2_-J questionnaire.

García-Aparicio and Herrero-Herrero [13] reported the original version of STCD_2_, in which the mean age of caregivers and patients with type 2 diabetes mellitus was 50 and 83 years, respectively. In contrast, the mean age of family caregivers in the present study was 64 years, and that of patients with diabetes mellitus was 72 years, which showed a tendency for family caregivers to be older. However, our findings that a higher proportion of family caregivers were female spouses and had chronic diseases and more patients were males were similar to those of a previous study on the original version [13]. The STCD_2_-J score in our study was 24.9, which was almost the same as that reported for the original version of the STCD_2_ questionnaire. However, as described above, caregivers were supporting older patients in the previous study by García-Aparicio and Herrero-Herrero [13], and caregivers in the previous study included formal caregivers. Therefore, the STCD_2_ scores should be interpreted considering the background characteristics of the caregivers and patients. In particular, among the questionnaire items in this study, scores for item 5, “satisfaction with own knowledge regarding diabetic treatment,” tended to be low. The percentage of family caregivers with experience in diabetes mellitus training and educational programs in this study was low (36%). The proportion of patients with complications associated with diabetes mellitus (retinopathy or nephropathy) in this study was also high, and 70% were insulin users. These findings suggest that family caregivers had fewer opportunities to participate in educational programs for diabetes mellitus or that complications in patients and frequent insulin use led to a decrease in the degree of satisfaction with the family caregivers' knowledge.

In this study, Cronbach's *α* coefficient was good at 0.88 for the STCD_2_-J questionnaire, and in the results of exploratory factor analyses, the STCD_2_-J questionnaire had a single-factor structure. Therefore, adding up the scores for STCD_2_-J questionnaire items would be appropriate to assess the degree of satisfaction of the family caregivers. In the criterion-related validation analysis, significant inverse correlations were found between the total and item-specific scores of the STCD_2_-J questionnaire and HbA1c, which were similar to the findings in the previous study conducted by García-Aparicio and Herrero-Herrero [13]. In the previous study, the most influential factor that increased the caregiver's degree of satisfaction was simplification of patients' medication (fewer medications or fewer administrations of medications) [13, 26]. In the present study, significant inverse correlations were also found between the number of injections administered to the patients and item 3 “satisfaction with the ease of injection and oral administration” (*r* = −0.30, *P* = 0.004) and item 4 “satisfaction with the number of treatments administered per day” (*r* = −0.34, *P* = 0.001), indicating the validity of the STCD_2_-J questionnaire. However, as in the previous study conducted by García-Aparicio and Herrero-Herrero [13], there were no significant associations between item 5 “satisfaction with own knowledge regarding antidiabetic treatment” of the STCD_2_-J questionnaire and HbA1c levels in this study. Results of previous studies have indicated that caregiver education or health literacy is highly related to glycemic control in such patients [27, 28]. The family caregivers' educational history or health literacy was not evaluated in this study, and it was unclear how these influenced the relationships between caregivers' satisfaction with their own knowledge and HbA1c levels. This is a point that necessitates further examination in the future. Results of previous studies have also revealed that decreased caregiver satisfaction is associated with mental health disorders, such as depression [9], nursing care burden and burnout [10, 11], and decreased QOL in patients [15]. Additionally, caregiver's psychological stress, depression, and negative self-assessment of nursing care contribute to difficulties in providing support [29] or to poor QOL for the caregiver [9]. In other words, the degree of satisfaction of the caregiver is closely related to mental health, acceptance of nursing care, and QOL. Based on these, the relationship between the STCD_2_-J scores and depression or negative self-assessment of nursing care was evaluated in this study, and significant inverse correlations were found. The validity of the STCD_2_-J questionnaire was considered to have been indicated even when it was assessed based on indicators other than the level of the glycemic parameter HbA1c in patients, including depression or self-assessment of the family caregiver regarding nursing care.

This study was the first to prepare the STCD_2_-J questionnaire and test its validity and reliability for use in family caregivers of elderly patients with diabetes mellitus requiring support in Japan. Furthermore, to the best of our knowledge, there are no family caregiver satisfaction assessment tools in Japan that have been formally back-translated or validated. As previously noted, the degree of satisfaction of family caregivers with patient treatment is associated with patient glycemic control [13, 14] and may also be associated with the family caregivers' own negative health outcomes. Therefore, considering the degree of satisfaction of family caregivers with patient treatment may be critical in the future, and gaining an understanding of the family caregivers' degree of satisfaction using the STCD_2_-J questionnaire may be clinically useful. The assumption is that the STCD_2_-J would be evaluated for family caregivers during an outpatient visit of dependent and elderly type 2 diabetic patients. For example, if the STCD_2_-J score is lower than the 25 point mean STCD_2_-J score obtained in this study, then the STCD_2_-J can be utilized as a trigger to provide support for family caregivers, such as simplifying drug treatment, providing diabetes training and education, or providing psychological support. In previous studies, it has been reported that the provision of education regarding caregiver knowledge and support skills or mental support improves caregiver's QOL [30–33]. There seems to be a need to evaluate the effects of simplifying drug treatments for patients with type 2 diabetes mellitus on the degree of satisfaction of family caregivers and their health outcomes or providing education and mental support regarding diabetes mellitus to family caregivers in the future.

This study has several limitations. First, our institution is a hospital with diabetes mellitus specialists, and there are numerous patients with relatively severe diabetes mellitus. Therefore, caution should be exercised when utilizing the STCD_2_-J questionnaire for family caregivers of patients with type 2 diabetes mellitus in other settings, such as in primary care. Second, because this was a cross-sectional study, it was not feasible to investigate how interventions altered the STCD_2_-J scores. In the future, changes in the STCD_2_-J scores will need to be verified in interventional studies. Third, the validation of cutoffs for STCD_2_-J scores for patient and family caregiver health outcomes was not feasible in this study and will require further investigation. Finally, the evaluation in this study may be inadequate because of its relatively small sample size. In the future, this evaluation must be repeated with a larger sample size.

## 5. Conclusions

In this study, the STCD_2_-J questionnaire was prepared, and its reliability and validity were analyzed. The results confirmed the reliability and validity of the STCD_2_-J questionnaire. In Japan, the STCD_2_-J questionnaire can be used as a tool to assess the satisfaction of family caregivers with respect to the treatment of elderly patients with type 2 diabetes mellitus requiring support.

## Figures and Tables

**Table 1:** Characteristics of the analysis population.

Family caregiver	
Age (years), mean (SD)	64.0 (13.4)
Male/female, *n*	55/153
Degree relative (%)	
Spouse	66.8
Parent	7.2
Children	21.1
Others	4.9
Occupation (%)	48.3
Duration of support (years), mean (SD)	10.7 (11.6)
Experience of the educational program (%)	36
Living with patient (%)	88.8
Numbers of diseases, mean (SD)	1.3 (0.4)
CACS (points), mean (SD)	20.4 (8.1)
PHQ-9 (points), mean (SD)	11.1 (4.2)
Patient	
Age (years), mean (SD)	72.5 (5.7)
Male/female, *n*	115/93
HbA1c (%), mean (SD)	7.1 (0.8)
OHA (%)	72.1
Administration times per day, mean (SD)	1.9 (0.8)
Insulin (%)	70.1
Administration times per day, mean (SD)	2.8 (1.2)
Retinopathy (%)	48.8
Nephropathy (%)	52.1
Cardiovascular disease (%)	31.8
DASC-8 (points), mean (SD)	13.1 (4.0)

SD: standard deviation; CACS: cognitive appraisal of caregiving scale; J-PHQ-9: Japanese version of the Patient Health Questionnaire-9; HbA1c: hemoglobin A1c; OHA: oral hypoglycemic agent; DASC-8: Dementia Assessment Sheet for Community-based Integrated Care System 8-items.

**Table 2:** Cronbach's alpha and factor loadings for STCD_2_-J.

Item no.	STCD_2_-J items	Mean (SD)	Alpha if item deleted	Factor loading
1	Are you satisfied with the treatment currently received by the person you are providing support or nursing care to?	3.8 (0.8)	0.85	0.82
2	Are you satisfied with the degree of acceptance of treatment by the person you are providing support or nursing care to?	3.7 (0.8)	0.85	0.8
3	Are you satisfied with the ease of treatment for the person you are providing support or nursing care to (e.g., injections and oral administration)?	3.7 (0.8)	0.85	0.81
4	Are you satisfied with the number of daily treatments received by the person you are providing support or nursing care to?	3.6 (0.8)	0.85	0.82
5	Are you satisfied with your knowledge of diabetes treatment?	3.1 (0.9)	0.89	0.45
6	Are you satisfied with the continuation of the same treatment?	3.6 (0.9)	0.85	0.81
7	Would you recommend to another person the treatment received by the person you are providing support or nursing care to?	3.2 (0.8)	0.88	0.53
	Total	24.9 (4.7)		

SD: standard deviation; STCD_2_-J: Japanese version of Satisfaction of Treatment among Caregivers of Dependent Type 2 Diabetic Patients.

## Data Availability

The data that support the findings of this study are available from the corresponding author upon request.

## References

[1] Centers for Disease Control and Prevention (2011). *National diabetes fact sheet: national estimates and general information on diabetes and prediabetes in the United States*.

[2] Ikeda N., Nishi N., Noda H., Noda M. (2017). Trends in prevalence and management of diabetes and related vascular risks in Japanese adults: Japan National Health and Nutrition Surveys 2003–2012. *Diabetes Research and Clinical Practice*.

[3] Sinclair A. J., Abdelhafiz A., Dunning T. (2018). An international position statement on the management of frailty in diabetes mellitus: summary of recommendations 2017. *Journal of Frailty and Aging*.

[4] Sinclair A. J., Armes D. G., Randhawa G., Bayer A. J. (2010). Caring for older adults with diabetes mellitus: characteristics of carers and their prime roles and responsibilities. *Diabetic Medicine*.

[5] Scarton L. J., Bakas T., Miller W. R., Poe G. D., Huber L. L. (2014). Needs and concerns of family caregivers of persons with type 2 diabetes: an integrated review of cross-cultural literature with implications for the American Indian population. *Diabetes Educator*.

[6] Schernthaner G., Schernthaner-Reiter M. H. (2018). Diabetes in the older patient: heterogeneity requires individualisation of therapeutic strategies. *Diabetologia*.

[7] Longo M., Bellastella G., Maiorino M. I., Meier J. J., Esposito K., Giugliano D. (2019). Diabetes and aging: from treatment goals to pharmacologic therapy. *Frontiers in Endocrinology*.

[8] Say R. E., Thomson R. (2003). The importance of patient preferences in treatment decisions—challenges for doctors. *BMJ*.

[9] Elstad E., Tusiofo C., Rosen R. K., McGarvey S. T. (2008). Living with Ma’I Suka: individual, familial, cultural, and environmental stress among patients with type 2 diabetes mellitus and their caregivers in American Samoa. *Preventing Chronic Disease*.

[10] Fredman L., Doros G., Cauley J. A., Hillier T. A., Hochberg M. C. (2010). Caregiving, metabolic syndrome indicators, and 1-year decline in walking speed: results of Caregiver-SOF. *Journals of Gerontology Series A, Biological Sciences and Medical Sciences*.

[11] Awadalla A. W., Ohaeri J. U., Al-Awadi S. A., Tawfiq A. M. (2006). Diabetes mellitus patients’ family caregivers’ subjective quality of life. *Journal of the National Medical Association*.

[12] Umegaki H., Yanagawa M., Nonogaki Z., Nakashima H., Kuzuya M., Endo H. (2014). Burden reduction of caregivers for users of care services provided by the public long-term care insurance system in Japan. *Archives of Gerontology and Geriatrics*.

[13] García-Aparicio J., Herrero-Herrero J. I. (2015). Development, validation, and administration of a treatment-satisfaction questionnaire for caregivers of dependent type 2 diabetic patients. *Clinical Interventions in Aging*.

[14] Bott U., Mühlhauser I., Overmann H., Berger M. (1998). Validation of a diabetes-specific quality-of-life scale for patients with type 1 diabetes. *Diabetes Care*.

[15] Awadalla A. W., Ohaeri J. U., Tawfiq A. M., Al-Awadi S. A. (2006). Subjective quality of life of outpatients with diabetes: comparison with family caregivers’ impressions and control group. *Journal of the National Medical Association*.

[16] Schwartz D. D., Cline V. D., Axelrad M. E., Anderson B. J. (2011). Feasibility, acceptability, and predictive validity of a psychosocial screening program for children and youth newly diagnosed with type 1 diabetes. *Diabetes Care*.

[17] Faulkner M. S., Clark F. S. (2016). Quality of life for parents of children and adolescents with type 1 diabetes. *The Diabetes Educator*.

[18] Japan Cabinet Office *Annual report on the aging society*.

[19] Vincent D., Clark L., Zimmer L. M., Sanchez J. (2016). Using focus groups to develop a culturally competent diabetes self-management program for Mexican Americans. *Diabetes Educator*.

[20] Strong C. (1984). Stress and caring for elderly relatives: interpretations and coping strategies in an American Indian and white sample. *Gerontologist*.

[21] Toyoshima K., Araki A., Tamura Y. (2018). Development of the Dementia Assessment Sheet for Community-based Integrated Care System 8-items, a short version of the Dementia Assessment Sheet for Community-based Integrated Care System 21-items, for the assessment of cognitive and daily functions. *Geriatrics and Gerontology International*.

[22] Muramatsu K. (2007). The patient health questionnaire, Japanese version: validity according to the mini-international neuropsychiatric interview-plus. *Psychological Reports*.

[23] Hirose M., Okada S., Shirasawa M. (2005). Structure of a scale to measure the cognitive assessment of family caregivers’ care–focusing on both positive and negative aspects. *Journal of Japan Academy of Home Care*.

[24] Yamamoto-Mitani N., Ishigaki K., Kuniyoshi M. (2004). Subjective quality of life and positive appraisal of care among Japanese family caregivers of older adults. *Quality of Life Research*.

[25] Raune D., Kuipers E., Bebbington P. E. (2004). Expressed emotion at first-episode psychosis: investigating a carer appraisal model. *British Journal of Psychiatry*.

[26] Lawton M. P., Brody E. M. (1969). Assessment of older people: self-maintaining and instrumental activities of daily living. *Gerontologist*.

[27] Hassan K., Heptulla R. A. (2010). Glycemic control in pediatric type 1 diabetes: role of caregiver literacy. *Pediatrics*.

[28] Reifegerste D., Hartleib S. (2016). Hypoglycemia-related information seeking among informal caregivers of type 2 diabetes patients: implications for health education. *Journal of Clinical and Translational Endocrinology*.

[29] Scarton L. J., Bakas T., Miller W. R., McLennon S. M., Huber L. L., Hull M. A. (2017). Development and psychometric testing of the diabetes caregiver activity and support scale. *Diabetes Educator*.

[30] Sörensen S., Pinquart M., Duberstein P. (2002). How effective are interventions with caregivers? An updated meta-analysis. *Gerontologist*.

[31] Kalra L., Evans A., Perez I. (2004). Training carers of stroke patients: randomised controlled trial. *BMJ*.

[32] Hendrix C. C., Bailey D. E., Steinhauser K. E. (2016). Effects of enhanced caregiver training program on cancer caregiver’s self-efficacy, preparedness, and psychological well-being. *Supportive Care in Cancer*.

[33] Alves S., Teixeira L., Azevedo M. J., Duarte M., Paúl C. (2016). Effectiveness of a psychoeducational programme for informal caregivers of older adults. *Scandinavian Journal of Caring Sciences*.

